# Pathology of Insanity

**Published:** 1857-04-01

**Authors:** W. Charles Hood

**Affiliations:** Resident Physician to Bethlehem Hospital


### Art. X.—PATHOLOGY OF INSANITY.

Based on the *Post-Mortem* Examinations in Bethlehem Hospital.

BY W. CHARLES HOOD, M.D.

Resident Physician to Bethlehem Hospital.

The following seven reports of post-mortem examinations represent, to a great extent, the mortality in Bethlehem Hospital during the years 1855 and 1856, and may be considered a continuation of similar reports previously communicated to the "Psychological Journal," by Dr. Webster. In the present series a short medical sketch of the patient has been added, with a view to render the cases more interesting, and possibly more instructive, than the bare extract from the Autopsy Book, without any clue to the psychological character of the case or treatment of the disease that had proved fatal.

Three of the following reports refer to criminal lunatics who died while under confinement in Bethlehem Hospital. Hitherto no reports have been made from this class of patients, owing to the restriction imposed by the authorities at the Home Office; but permission has recently been granted by the Secretary of State for the Home Department to include in this and future autopsies, reports of those cases of criminal lunatics who die in the hospital.

1. T. C. P., a male criminal lunatic; resident in the hospital nineteen years; died aged 55. Had been an idiot from childhood; during his residence in the hospital his behaviour was generally quiet and tractable, but subject to occasional paroxysms of excitement, when he became destructive, noisy, and dirty in his habits. These attacks lasted a few weeks, and were followed by months of tranquillity: he was very deaf; had great difficulty in articulation, his language being little more than a noisy jabber of incoherent sounds. His habit of body was most obese, and his temperament phlegmatic; his physical health was generally good, requiring little medicine except aperients, but those of a drastic character. On the 3rd of June, 1856, he was attacked with erysipelas of the face and scalp, from which he died on the 13th of the same month.

*Autopsy.*—The skull-cap very thick, and of small dimensions; posteriorly very broad. The brain small but well developed, of firm consistence, and the parts at the base clearly distinct. The ophthalmic arteries were loaded with atheromatous deposit, and the same condition, in a less degree, was observed in the basilar artery and the other arteries at the base of the brain.

The abdominal parietes consisted chiefly of a great thickness of fat; the intestines were loaded with fat, and the muscular colour of the heart concealed by its being imbedded in adipose tissue. The thoracic and abdominal viscera were all healthy, with the exception of slight old adhesions in the anterior part of the right pleura.

2. C. S., a male criminal lunatic, who voluntarily confessed a murder he had perpetrated; was tried, convicted, and acquitted on the ground of insanity. Resident in the hospital three years, and died of exhaustion following acute mania of one week's duration. Previous to this attack of mania, which came on without any observable exciting cause, he had been quiet and inoffensive, avoiding the company of others, and refusing all conversation with his companions. On three occasions during the last year of his life he obstinately refused his food, until it was considered necessary to administer it to him with the stomach pump. To the operation he submitted without opposition, and at the next meal readily took his food.

*Autopsy.*—Fifty hours after death.—Body emaciated; several bruises and small scars over various parts; skull-cap rather thin, especially posteriorly; brain soft; membranes apparently healthy; lungs, heart, kidneys, healthy. There was a tumour about the size of a goose's egg attached to the fascia, and lying on the left side between the internal edge of the psoas muscle and the external border of the left iliac artery, apparently of a cartilaginous character.

3. J. R., a female patient, aged fifty, died of phthisis, having been resident in the hospital eleven months. On admission she was suffering from mania, with delusions. Her conversation was incoherent and rambling, frequently and abruptly commencing singing in behalf of the rights of women and children. Her delusions consisted in a belief that her inside was composed of iron and steel, for which reason she was to be divorced from her husband. During the first two months her excitement was to some extent governable by change of room or occupation, and allowing her to roam about the airing ground unrestricted by the company of other patients. Subsequently she became more and more violent, mischievous, and destructive; her habits dirty in the extreme; conversation filthy; and she continued in this state to the day of her death. During the early stage of her disease, the excitement was in some measure allayed by tartarized antimony, in half-grain doses three times a day; the effect was only temporary, and no medicine of either a sedative or depressant character afforded any permanent benefit. During the long period of excitement she was sustained by a generous diet, without stimulants.

*Autopsy.*—Nearly all the parts, both external and internal, were in a highly anæmic state. The blood-vessels of the head, both external and internal, were empty. The cellular tissue of the scalp and face was infiltrated with a semi-fluid and slightly opaque effusion. The cerebral substance generally soft, but without morbid change; a little fluid in the lateral ventricles. The left lung healthy in structure, connected to the parietal pleura by general old and strong adhesions. The right lung presented one adhesion only, of small extent, the rest of the surface being quite free. It was healthy in structure, except at the posterior part of each lobe, in which there had been inflammation, with congestion, partial consolidation, and suppuration. The matter, thick, healthy in appearance, and free from unpleasant smell, was collected in small abscesses. There was one in the upper lobe, containing about a teaspoonful of pus, and three in the lower lobe, from which a tablespoonful flowed. The pus in each abscess had advanced to the surface, so as to be visible by its yellow colour; but the lung had not become adherent.

No diseased change was observed in the abdomen, from which, as well as from the thorax, all adipose structure had disappeared.

4. B. D., a female patient, aged thirty-one; married; duration of attack before admission, six weeks. Died of carbuncle, having been resident in the hospital four months. On admission, she was suffering from acute mania, with intermittent paroxysms of great violence. Before being brought to the hospital, her insanity had been evinced by great and unusual irritability of temper; violently refusing to take food, go to bed, dress or undress herself, remain in her room or in the house, unless forcible means were adopted to oblige her. For the first three months after admission there was but little change or improvement in her symptoms. She remained on alternate days obstinate, irritable, taciturn, and violent; was compelled to take her food with great difficulty, and required constant attention. On other days, her mental state appeared to undergo a perfect revolution. She rose in the morning cheerful, good-tempered, industrious, and, to a casual visitor, she was convalescent. During the fourth month her mental state steadily and permanently improved, but her physical strength sank with the exhaustion following carbuncles.

*Autopsy.*—Considerable general emaciation; the skull-cap heavy; partial milky white opacity of the arachnoid over the entire convexity of both cerebral hemispheres; slight infiltration of the pia mater; about three ounces of limpid fluid in the lateral ventricles; the foramen of Monro converted into a large direct opening between the two cavities; the bloody points in section of the cerebral substance numerous and large; some ounces of dropsical fluid in the pericardium; concentric hypertrophy of the left ventricle of the heart, of which the muscular substance was firm, and the cavity small; the liver and kidneys healthy.

5. W. C., a male criminal lunatic, acquitted, on the ground of insanity, of the crime for which he was tried; thirty years of age; of a low, melancholy temperament, dissatisfied with everybody and everything. His occupation had been that of a sailor; his habits of life intemperate. He suffered much from disease of the spine, occasioned by a fall from the rigging of his ship; his head was not hurt at that time, nor was his mind observed to be diseased until many years after; his insanity was principally marked by moroseness and irritability; he was dangerous to others, frequently striking people without other provocation than a fancy that they intended to annoy him. On the evening of the day of his death, he was sitting engaged with one of his companions playing a game of chess, when he suddenly left the table for the closet, intimating his intention of returning immediately. A quarter of an hour elapsed without his doing so; and when search was made for him, he was found hanging by his braces to the lintel of the door;—the body was warm, but life quite extinct.

*Autopsy.*—The contents of the cranium healthy, except very slight and almost doubtful partial opacity of the arachnoid, and a few drachms of fluid in the lateral ventricles; the left lung adhering to the cavity universally, and so strongly that the substance was extremely lacerated in attempting to detach it; two or three partial adhesions on the right side. Behind the posterior mediastinum, and extending from the second to the tenth dorsal vertebræ, there was a collection of well-formed pus in the front of the spinal column; it was contained in a thick sac of irregular and sacculated surface, closely adherent to the sides of the column. The bodies of the vertebræ at the middle of this space were bare here and there to a small extent. This collection of matter corresponded to an angular curvature on the posterior aspect of the affected spinal region, ascribed by the patient to a fall from the rigging to the deck of a vessel. In the upper part of the abdomen, the viscera, apparently healthy, were closely adherent to each other and to the walls of the abdomen. The convex surface of the liver adhered throughout to the diaphragm, while its concavity was so closely connected to the stomach that careful dissection would have been required to separate them without injury. The stomach adhered to the abdominal walls and to the colon. The upper convolutions of the small intestine were partially connected to the colon and mesocolon, as well as to each other, by thin and elongated adhesions. No other morbid appearance was observed in the abdomen.

6. S. C., a male patient, died of phthisis, having been under treatment in the hospital three months. This patient had been a publican by trade, and formerly was well to do in the world; but intemperate habits injured his business, and the consequent loss of property caused serious mental excitement. On admission he was excited, and, if restrained, violent to any one who came in his way. He fancied himself ill-used, and on that account threatened to cut his throat and make away with himself. His conversation was incoherent and rambling. Morphia was given in grain doses three times in the day, but without any beneficial effect, and after three weeks was discontinued. His paroxysms of excitement caused deep after-depression, and his bodily health rapidly sank, with cough, hectic, and all the symptoms of pulmonary disease.

*Autopsy.*—Examination made thirty hours after death. Body emaciated. The contents of the skull healthy; nothing remarkable in their appearance. In the right pleura there was about a pint of opaque milky fluid, and the lung itself was externally of the same colour, flattened, and much decreased in size. At the apex were two or three vomicæ containing purulent fluid; and throughout the structure of the organ tuburcles were thickly scattered; the structure itself was hepatized, and appeared to contain no air; there was considerable long-standing adhesion. The left lung was perfectly healthy. Liver large and healthy; gall-bladder very distended with bile. Kidneys smaller than natural: on the surface of the right one a small hard tuburcle lying beneath the capsule, and a small cyst about the size of a mustard-seed in the same position on the left kidney. Intestines healthy.

7. H. G. H., a female patient, died from the exhaustion following acute mania; the mental disease had existed four days previous to her admission. When brought to the hospital, she was perfectly unconscious, and in a state of such extreme exhaustion, that it was necessary at once to place her in bed, and employ every measure to bring about reaction. From the report of her friends it appears she had taken little or no nourishment for some days previous to the commencement of the attack. She lingered on, just sufficiently conscious to swallow liquid nourishment if placed in her mouth, but ultimately sank on the fifth day after admission.

*Autopsy.*—The brain and its membranes healthy, the only circumstance observed within the head being a slightly increased quantity of fluid in the base of the skull after the brain had been removed.

The heart appeared considerably larger than usual. This depended entirely upon great distension of the right cavities with blood. The right lung was healthy, as was also the upper lobe of the left. The posterior lobe of the left, at its posterior aspect, was in the congestive stage of pneumonia; on cutting through this part and squeezing it, a small quantity of thickish puriform fluid escaped at two or three points. The abdominal viscera were healthy.

*(To be continued.)*

---

